# Tfh cell subset biomarkers and inflammatory markers are associated with frailty status and frailty subtypes in the community-dwelling older population: a cross-sectional study

**DOI:** 10.18632/aging.102789

**Published:** 2020-02-08

**Authors:** Ming-Juan Yin, Yong-Zhen Xiong, Xiu-Juan Xu, Ling-Feng Huang, Yan Zhang, Xiao-Jun Wang, Liang-Chang Xiu, Jing-Xiao Huang, Ting-Yu Lian, Dong-Mei Liang, Jin-Mei Zen, Jin-Dong Ni

**Affiliations:** 1 Department of Preventive Medicine, Dongguan Key Laboratory of Environmental Medicine, School of Public Health, Guangdong Medical University, Dongguan, China; 2 School Clinic, Guangdong Medical University, Dongguan, China; 3 Department of Epidemiology and Biostatistics, Dongguan Key Laboratory of Environmental Medicine, School of Public Health, Guangdong Medical University, Dongguan, China

**Keywords:** frailty status, frailty subtypes, biomarkers, community-dwelling older population

## Abstract

We conducted a cross-sectional study investigating community-dwelling older population to determine association between immunoscenescence marker, inflammatory cytokines and frailty. Frailty status was classified with 33-item modified frailty index and latent class analysis was applied to explore the latent classes (subtypes) of frailty. In multivariable analysis, higher Tfh2 cells were associated with a higher risk of frailty [1.13(1.03–1.25)] in females, but a lower risk of cognitive and functional frail [0.92(0.86–0.99)] and physiological frail [0.92(0.87–0.98)]. Additionally, a greater risk of multi-frail and physiological frail correlated with low Tfh1 [0.77(0.60–0.99); 0.87(0.79–0.96)] and Tfh17 cells [0.79(0.65–0.96); 0.86(0.78–0.94)], respectively. Higher B cells were associated with decreased frailty/pre-frailty both in females [0.89(0.81–0.98)] and males [0.82(0.71–0.96)], but did not correlate with frailty subtypes. Regarding inflammatory markers, participants in the TGF-β 2^nd^ quartile showed a decreased risk of pre-frailty/frailty in females [0.39(0.17–0.89)] and psychological frail [0.37(0.16–0.88)], compared with those in the top tertile. Moreover, we found participants in the 2^nd^ tertile for IL-12 levels showed a decreased risk of physiological frail [0.40 (0.17–0.97)]. Our study highlights the importance of Tfh cell subsets and inflammatory markers in frailty in a sex-specific manner, particularly in terms of frailty subtype.

## INTRODUCTION

Frailty, which is highly prevalent among the older population, is generally characterized by decreased functional and physiological reserves, ultimately resulting in a higher incidence of disability, comorbidities and even mortality when exposed to stress than among age-matched counterparts [1]. With a growing aging population and the increasing health-related burden, there is increased emphasis on age-related frailty worldwide, which has emerged as a concept with which to explore the health trajectory of older subjects, especially in developed countries. However, the complex underlying pathophysiological biomarkers for this condition have not been well-established.

With regard to frailty, a robust diagnosis in a clinical setting is a precondition for frailty research. However, there is currently no consensus on the standardized criteria or operational definition for the measurement of frailty, which has hindered the development of biomarkers for this condition. Most of the current frailty screening tools are based on two complementary rather than substitutive models including phenotype model [1] and deficit model [2] whose application is mainly dependent on the specific clinical settings. Fried phenotype by Fried [1] which is based on five criteria primarily capture declines in physical functions. However, assessment of some components such as grip strength and gait speed needs special equipment and is time consuming, limiting its applicability in multiple settings. During the same period, the frailty index (FI) proposed by Rockwood [2] accumulated deficits in multiple domains is generally considered to be the more appropriate tool available to identify prefrailty or frailty. Taking ethnicity and societal factors into consideration, a modified FI (mFI) model was developed to recognize progression from non-frail or prefrail to frail status in the older Chinese population by our team several years ago [3]. However, frailty is complex and heterogeneous and the FI score did not completely reflect how each individual component was clustered [4, 5]. Furthermore, some researchers began to explore physical frailty [5] and cognitive frailty [6] subtypes, which led to the idea that unique components could be classified to targeted interventions in older individuals by subtype analysis.

Currently, several biomarkers have been proposed for frailty, which involve multiple physiological systems, including, most prominently, immune system alterations and inflammatory states [7]. Age-related immunosenescence due to thymic involution has been widely used to describe immune system alterations, which could not only result in negative outcomes such as susceptibility to infection or vaccine failure [8] but also contribute to many aging-associated conditions such as frailty in older adults [9]. Of all immunosenescence compartments, increasing evidence reveals that the adaptive immune system, especially T cell phenotype, undergoes remodeling with advancing age and suggests its important role in frailty [10]. Substantial evidence supportive of a role of T cell subset alterations the pathogenesis of frailty has also emerged in different populations, including the general population [11, 12] and HIV-infected patients [13]. Recently, follicular helper T (Tfh) cells, characterized by high expression of CXCR5, PD-1 and ICOS, have been clarified as a critical subset of T cells in infections via a stable interaction with B cells [14]. Tfh cells can be divided into three subpopulations: Tfh1 (CXCR3^+^CCR6^−^), Tfh2 (CXCR3^−^CCR6^−^) and Tfh17 (CXCR3^−^CCR6^+^) cells according to the expression of CXCR3 and CCR6 [15]. Interestingly, specific Tfh cell polarization emerged after challenge with different antigens and different subsets displayed distinct capacities to assist B cells [16]. It is obvious that Tfh and B cell alternation with aging directly affects the adaptive immune system state, which further reduced the capacity to cope with impairment [17]. However, Tfh cell and subset polarization and immune dysregulation in frail older individuals are poorly understood [18].

Besides immune senescence, substantial evidence indicates that heightened inflammatory state alterations also play an essential role in the pathogenesis of frailty. Some inflammatory markers have been found to be associated with frailty in older subjects in general [19], Alzheimer's disease patients [20] and cancer patients [21] in previous studies. Furthermore, cytokines produced by cells of the adaptive immune system are important for inflammatory processes [22]. Accordingly, investigating the available role of cytokines in Tfh cell activation and function [23], along with aging or frailty processes [24, 25], might reveal new insights into inflammatory markers associated with frailty without single or traditional markers.

To date, the association between specific T cell subset markers of immune differentiation and senescence and frailty status in older individuals is based on only limited evidence of memory/naïve CD4^+^ T cells, CD8^+^ T cells or regulatory T cells [11, 12, 26, 27]. Previous frailty studies have mainly focused on well-known inflammation markers, such as IL-6, CRP and TNF-α [28, 29], some with conflicting results [28, 30]. In addition, immune senescence rates differ between the sexes, with the female sex being an important factor predisposing individuals to frailty [31–33]. However, previous reports did not consistently demonstrate an association between frailty and sex-specific T cell subsets or inflammation markers. Furthermore, the emergence of frailty subtypes may reveal specific patterns of underlying factors in different domains. Taking these into consideration, we performed a cross-sectional analysis to explore the association between Tfh cells and subsets, and proposed inflammatory biomarkers with frailty status and frailty subtypes as defined by mFI in the older Chinese population with a special focus on sex-related differences. Our findings provided evidence that timely targeted intervention could delay the onset of late-life poor outcomes in non-frail or pre-frail older individuals.

## RESULTS

### Frailty classification

### Frailty according to the modified frailty index and frailty subtypes

Based on mFI, the community-dwelling older individuals enrolled in the study were categorized into three frailty states (n=689) with 31.8% classified as non-frail, 51.7% as pre-frail and 16.5% as frail. As expected, the median of mFI for frailty status was 0.046 (interquartile range, IQR, 0.030–0.061), 0.136 (IQR, 0.106–0.167) and 0.303 (IQR, 0.242–0.394), respectively. Furthermore, females had a higher mFI score than males in the frailty group [0.318 (IQR, 0.242–0.424) vs. 0.273 (0.235–0.364), *P*<0.001] (Table 1).

**Table 1:** Characteristics of the study population according to frailty status by modified frailty index.

**Characteristic**	**Non-frail**	**Pre-frail**	**Frail**	***χ^2^***	***P* value**
**N, %(n)**	31.8% (219)	51.7% (356)	16.5% (114)	-	*-*
**Age, %(n)**					
60-69	52.1% (114)	36.0% (128)	23.7% (27)	86.351	<0.001
70-74	23.7% (52)	31.6% (109)	16.7% (19)		
75-79	16.4% (36)	17.4% (62)	16.7% (19)		
80-84	6.8% (15)	10.7% (38)	22.8% (26)		
≥85	0.9% (2)	5.3% (19)	20.2% (23)		
**Sex, %(n)**					
Female	53.4% (117)	65.7% (234)	76.3% (87)	18.448	<0.001
Male	46.6% (102)	34.3% (122)	23.7% (27)		
**Education, %(n)**					
Illiterate	10.6% (23)	25.1% (88)	45.6% (52)	55.331	<0.001
Primary	66.8% (145)	58.4% (205)	48.2% (55)		
Secondary and above	22.6% (49)	16.5% (58)	6.1% (7)		
**Smoking, %(n)**					
Smoking	23.8% (48)	17.2% (60)	8.4% (9)	29.387	<0.001
Smoking in the past	22.8% (46)	16.4% (57)	7.5% (8)		
Never	53.5% (108)	66.4% (231)	84.1% (90)		
**mFI, median (IQR)**	0.046 (0.030-0.061)	0.136 (0.106-0.167)	0.303 (0.242-0.394)	-	-
**mFI (Females), median (IQR)**	0.061 (0.030-0.061)	0.136 (0.106-0.182)	0.318 (0.242-0.424)	359.998	<0.001
**mFI (Males), median (IQR)**	0.046 (0.030-0.061)	0.136 (0.106-0.167)	0.273 (0.235-0.364)	204.508	<0.001

N=689. All results reported as % (n) or median (IQR). Differences between frailty groups determined by Pearson’s χ^2^ test or Kruskal-Wallis text. *P* < 0.05. Abbreviations: IQR: interquartile range.

Variables with missing values: Education (7/689; 1.0%), Smoking (32/689; 4.6%).

When measuring frailty subtypes (n=728), the study extracted one to seven potential class models and the fitting results are shown in Supplementary Table 1. As the model classification increased from one to seven, the values of the Lo–Mendell–Rubin (LMR) and the bootstrap-based likelihood ratio test (BLRT) reached significant levels (*P*<0.01) when five categories were retained. Therefore, the classification using five latent classes (Class 1, Class 2, Class 3, Class 4 and Class 5) was finally selected according to the latent class model fitting results. Further, we distinguished five latent classes, including “relatively healthy” (51.6%) and four frailty subtypes, namely “multi-frail” (2.3%), “cognitive and functional frail” (9.8%), “psychologically frail” (15.3%) and “physiologically frail” (21.0%) (Supplementary Table 2). Detailed information regarding the naming of latent classes is shown in the Supplemental Information.

### Relationship between the modified frailty index and frailty subtypes

**Figure 1:**
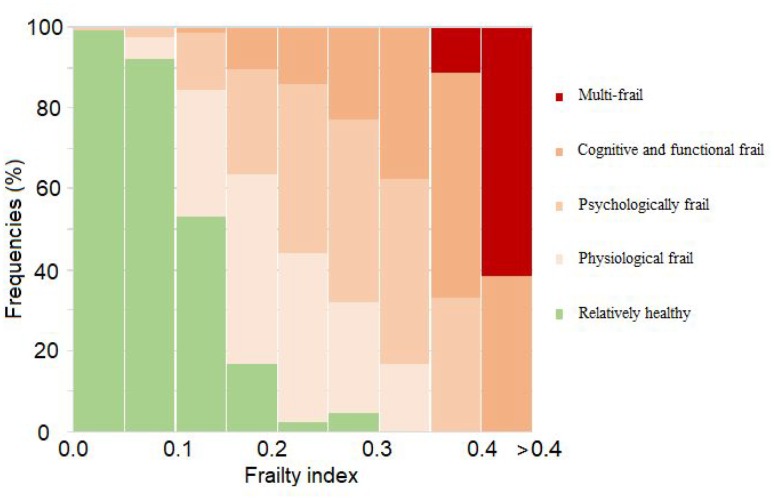
**Relationship between modified frailty index and frailty subtypes.**

We explored the relationship between the mFI score and frequencies for each frailty subtype. The results showed that when mFI increased (range from 0 to 0.3), the proportion classified as relatively healthy decreased at a frequency of more than 50% (range from 0 to 0.15). The proportion classified as cognitive and functional frail (range from 0.05 to 0.35), psychologically frail (range from 0 to 0.4) and physiologically frail (higher than 0.1), increased then decreased along with an increased mFI score. When the mFI score was higher than 0.4, the multi-frail classification gradually became predominant (Figure 1).

### Participant characteristics

Of 728 participants, the mean age was 73.09±7.85 years (range 63–120) and female participants accounted for 64%. The main baseline characteristics of the participants in the three frailty status groups are shown in Table 1. Frail participants (77.32±7.54 years) were older than pre-frail (72.63±8.09 years) (*P*<0.001) and non-frail participants (70.90±5.03 years) (*P*<0.001). Female participants accounted for 66% and 76% in the pre-frail and frail groups, respectively. There were also significant differences in education level and smoking between the frailty groups (*P*<0.001).

### Sex-related differences in the association between biomarkers and frailty status

**Figure 2:**
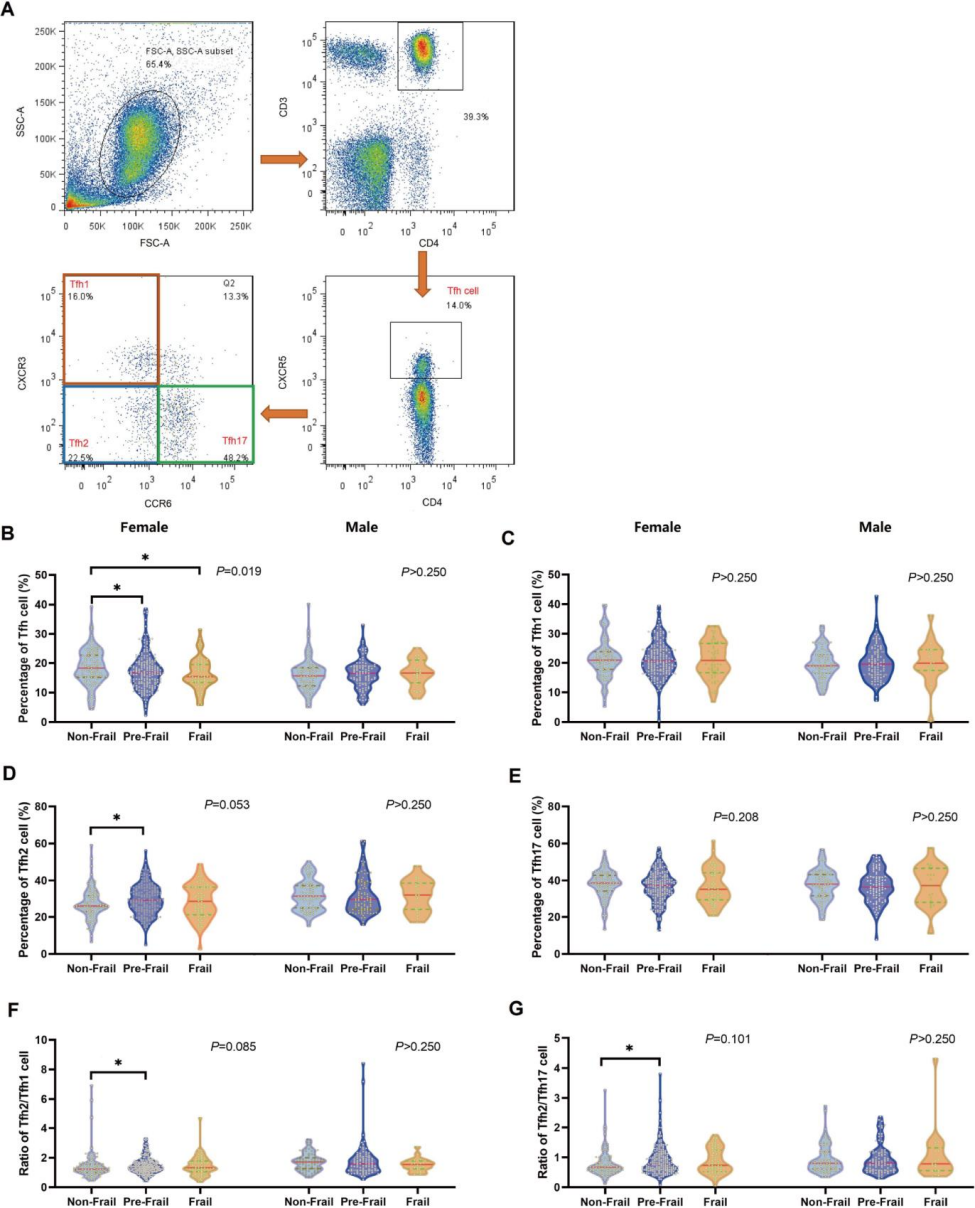
**Comparison of the Tfh cell and subsets phenotype distributions in the CD4^+^T cell in old individuals categorized with modified frailty index.** (N=689) (**A**) Gating strategy for Tfh cells and subsets. Representative sample is shown and numbers indicate population frequency. Comparison of the (**B**) Tfh cell, (**C**) Tfh1 cell, (**D**) Tfh2 cell, (**E**) Tfh17 cell, (**F**) ratio of Tfh2/Tfh1 cell and (**G**) ratio of Tfh2/Tfh17 cell proportions in the CD4^+^T cell by frailty groups in both female and male. *P < 0.05.

**Figure 3:**
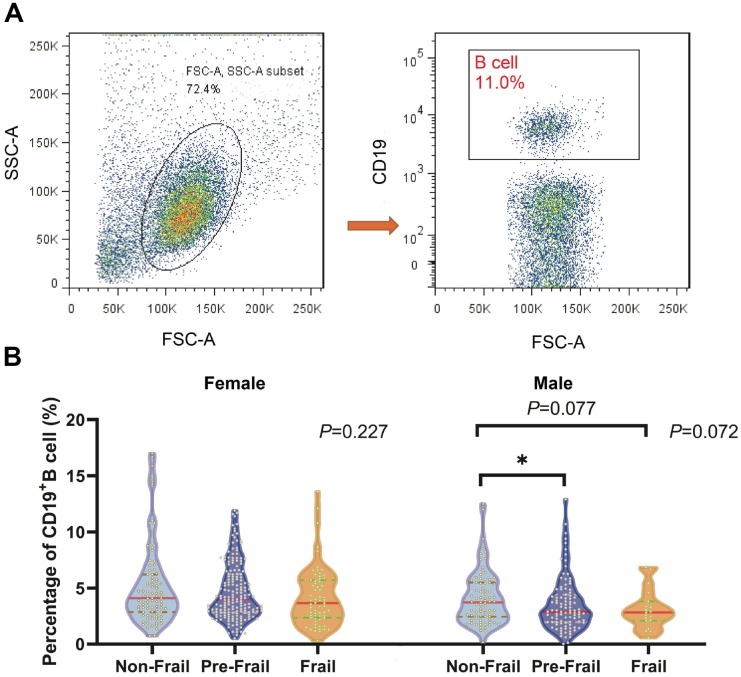
**Comparison of the CD19^+^ B cell proportions in the lymphocyte in old individuals categorized with modified frailty index.** (N=689) (**A**) Gating strategy for CD19^+^ B cells. Representative sample is shown. (**B**) Comparison of the median CD19^+^ B cell proportions in the lymphocyte of different frailty group in both female and male. *P < 0.05.

Firstly, we analyzed immunosenescence markers in the different frailty status groups for both sexes. The gating strategy for Tfh cells and subsets is shown in Figure 2A. With regard to the Tfh cell and subset proportions, both pre-frail (*P*<0.05) and frail participants (*P*<0.05) showed significantly lower levels of Tfh cells than non-frail participants (Figure 2B), while Tfh2 cells were significantly higher in pre-frail participants than in non-frail participants (*P*<0.05) (Figure 2D), although this was only the case in females, not in males. Similarly, significantly higher Tfh2/Tfh1 cell (*P*<0.05) and Tfh2/Tfh17 cell (*P*<0.05) ratios were also observed in pre-frail participants compared with non-frail participants in females (Figure 2F, 2G). The proportions of CD19^+^ B cells significantly differed between the groups for both females (*P=*0.227) and males (*P=*0.072) and were significantly lower in pre-frail than in non-frail participants in males (*P*<0.05) (Figure 3).

Next, we analyzed the expression of inflammatory markers, including IL-6 [28], IL-12 [24], TGF-β [25] and Tfh-secreted IL-21, in the different groups after comprehensive assessment. These markers were investigated for each quartile for the univariate analyses for both sexes. Significant differences were observed in TGF-β (*P*=0.137) and IL-6 (*P*=0.075) levels in females, and IL-12 (*P*=0.088) in males, between non-frail, pre-frail and frail individuals (Table 2).

**Table 2:** Characterristics of inflammatory markers according to frailty status by modified frailty index in both female and male.

**Inflammatory markers**	**Non-frail**	**Pre-frail**	**Frail**	***χ^2^***	***P* value**
**Female**					
**TGF-β, % (n)**					
1st	23.3% (27)	24.4% (57)	14.9% (13)	9.721	0.137*
2nd	23.3% (27)	23.5% (55)	21.8% (19)		
3rd	30.2% (35)	26.5% (62)	23.0% (20)		
4th	23.3% (27)	25.6% (60)	40.2% (35)		
**IL-6, % (n)**					
1st	21.4% (25)	21.4% (50)	19.5% (17)	11.459	0.075*
2nd	27.4% (32)	28.6% (67)	12.6% (11)		
3rd	24.8% (29)	21.4% (50)	32.2% (28)		
4th	26.5% (31)	28.6% (67)	35.6% (31)		
**IL-12, % (n)**					
1st	21.6% (25)	25.7% (59)	18.8% (16)	3.023	0.806
2nd	26.7% (31)	23.9% (55)	30.6% (26)		
3rd	25.9% (30)	25.2% (58)	22.4% (19)		
4th	25.9% (30)	25.2% (58)	28.2% (24)		
**IL-21, %(n)**					
1st	19.7% (23)	26.9% (63)	21.8% (19)	3.053	0.802
2nd	25.6% (30)	24.4% (57)	23.0% (20)		
3rd	25.6% (30)	24.4% (57)	26.4% (23)		
4th	29.1% (34)	24.4% (57)	28.7% (25)		
**Male**					
**TGF-β, %(n)**					
1st	28.4% (29)	30.0% (36)	29.6% (8)	6.573	0.362
2nd	22.5% (23)	21.7% (26)	22.2% (6)		
3rd	22.5% (23)	23.3% (28)	40.7% (11)		
4th	26.5% (27)	25.0% (30)	7.4% (2)		
**IL-6, %(n)**					
1st	30.4% (31)	23.8% (29)	33.6% (9)	3.352	0.764
2nd	20.6% (21)	28.7% (35)	25.9% (7)		
3rd	22.5% (23)	23.8% (29)	22.2% (6)		
4th	26.5% (27)	23.8% (29)	18.5% (5)		
**IL-12, %(n)**					
1st	30.0% (30)	27.1% (32)	42.3% (11)	11.009	0.088*
2nd	29.0% (29)	16.9% (20)	26.9% (7)		
3rd	19.0% (19)	28.8% (34)	23.1% (6)		
4th	22.0% (22)	27.1% (32)	7.7% (2)		
**IL-21, %(n)**					
1st	31.4% (32)	27.9% (34)	33.3% (9)	2.585	0.859
2nd	25.5% (26)	23.0% (28)	25.9% (7)		
3rd	23.5% (24)	21.3% (26)	22.2% (6)		
4th	19.6% (20)	27.9% (34)	18.5% (5)		

N=689. Inflammatory markers levels are as follows: 1st quartile, 2nd quartile, 3rd quartile, 4th quartile. “*” indicate significant differences between frailty groups and inflammatory marker entering ordinal logistic regression models. Variables with missing values: IL-12 (14/689; 2.0%), TGF-β (3/689; 0.4%).

**Figure 4:**
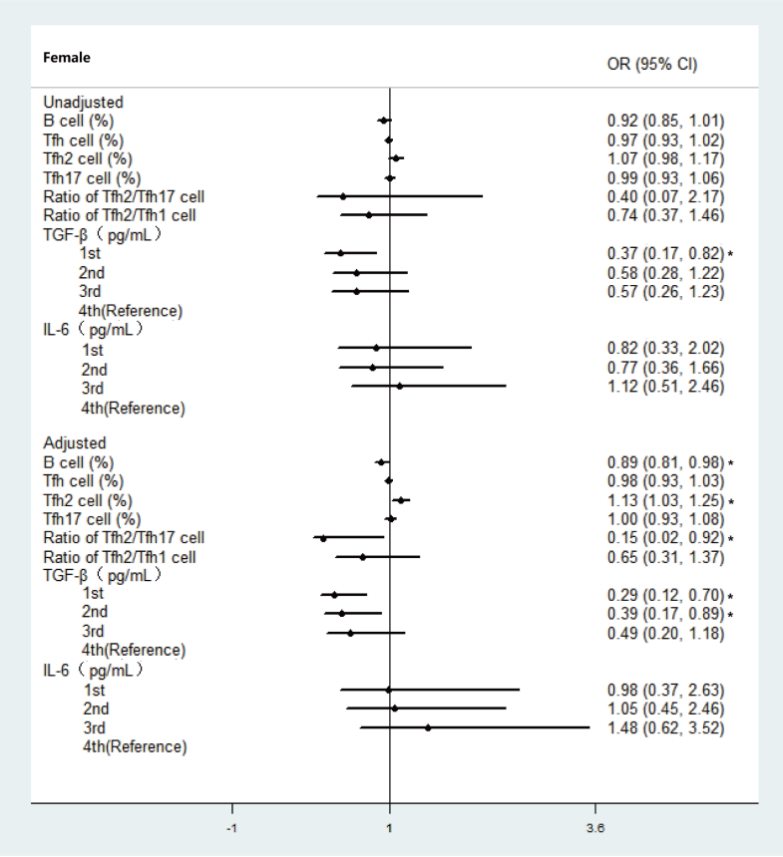
**Ordinal logistic regression analysis between immune parameter and frailty group categorized with modified frailty index in female.** Abbreviations: OR, Odds ratio; CI, confidence interval; *P < 0.05

**Figure 5:**
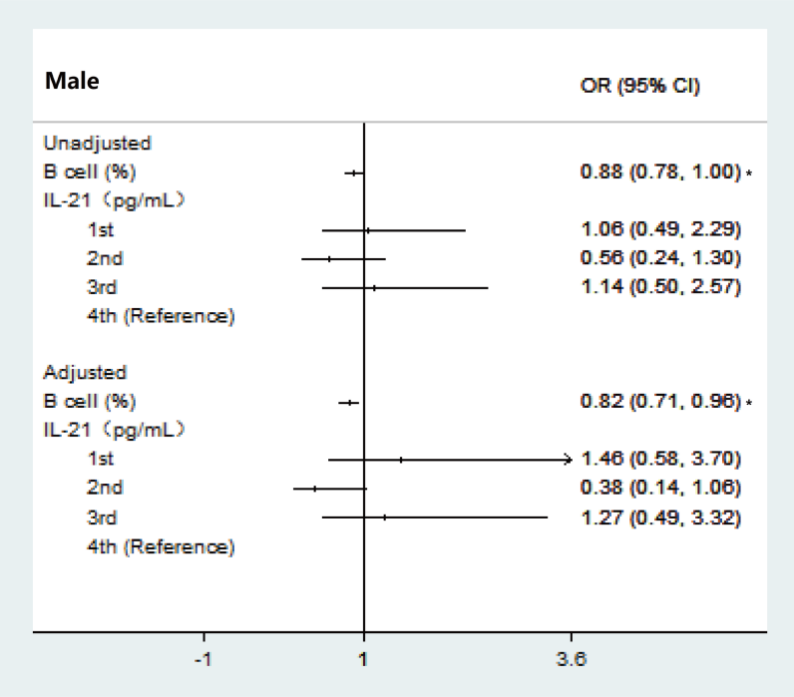
**Ordinal logistic regression analysis between immune parameter and frailty group categorized with modified frailty index in male.** Abbreviations: OR, Odds ratio; CI, confidence interval; *P < 0.05

Then, biomarkers with *P*<0.25 were included in the final ordinal logistic regression analysis. As shown in Figure 4, higher percentages of Tfh2 cells were associated with a higher risk of frailty (adjusted odds ratio (aOR) = 1.13; 95% confidence interval (CI), 1.03–1.25) in females. By contrast, a higher ratio of Tfh2/Tfh17 cells was more likely to be associated with a lower frailty level (aOR = 0.15; 95% CI, 0.02–0.92). Higher percentages of B cells showed decreased odds of being frail or pre-frail both in females (aOR = 0.89; 95% CI, 0.81–0.98) and males (OR = 0.82; 95% CI, 0.71–0.96) (Figures 4 and 5). In terms of inflammatory markers, being in the bottom quartile for TGF-β was associated with a lower risk of frailty in females (OR = 0.37; 95% CI, 0.17–0.82) compared with the top quartiles. After adjusting for baseline characteristics in the multinomial regression model, participants with TGF-β levels in the bottom and 2nd tertiles had a lower risk of being pre-frail or frail, compared with those in the top tertile in females (aOR = 0.29; 95% CI, 0.12–0.70; aOR = 0.39; 95% CI, 0.17–0.89), whereas no significant association in the frailty status was observed across tertiles for IL-6 (Figure 5).

### Association between biomarkers and frailty subtypes

**Figure 6:**
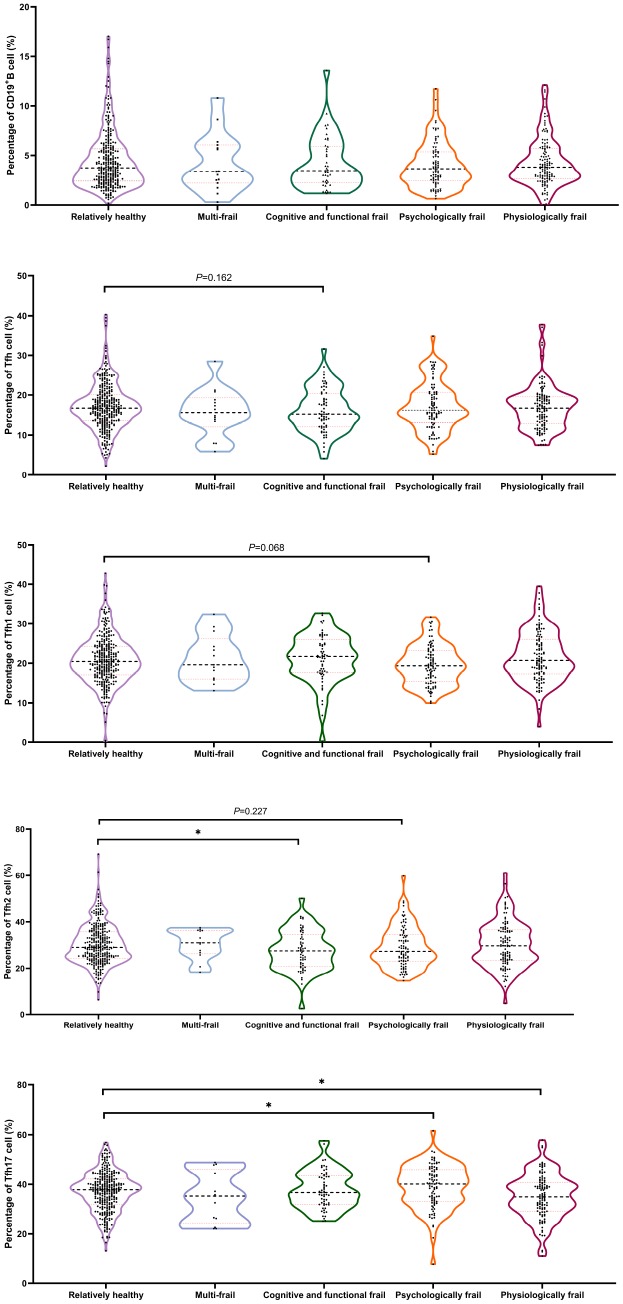
**Comparison of the Tfh cell and subsets proportions in the CD4^+^T cell in old individuals categorized with frailty subtypes.** (N=728) “Relatively healthy” as reference; *P < 0.05.

With regard to immunosenescence markers, cognitively and functionally frail participants showed a significantly lower percentage of Tfh (*P* = 0.162) and Tfh2 (*P*<0.05) cells than relatively healthy subjects, whereas Tfh1 cells were significantly lower in psychologically frail individuals than in relatively healthy participants (*P* = 0.068). Significantly lower levels of Tfh17 cells were observed in physiologically frail individuals compared with relatively healthy participants (*P*<0.05), and in psychologically frail compared with relatively healthy participants (*P*<0.05). No significant difference in the frailty subtype was observed in terms of the B cell profile (Figure 6). Significant differences were observed; however, in TGF-β levels in multi-frail and cognitive and functional frail individuals compared with relatively healthy participants, and in IL-12 and IL-21 levels between cognitive and functional frail individuals and relatively healthy participants (Table 3).

**Table 3:** Characterristics of inflammatory markers according to frailty subtypes (“Relatively healthy” as reference).

**Immune parameter**	**Multi-frail**	**Cognitive and functional frail**	**Psychologically frail**	**Physiologically frail**	**Relatively healthy**
**TGF-β, %**					
1st	0.0%	20.0%	26.6%	21.6%	28.8%
2nd	29.4%	24.3%	23.9%	26.1%	23.7%
3rd	23.5%	21.4%	22.9%	26.8%	25.3%
4th	47.1%	34.3%	26.6%	25.5%	22.1%
***χ^2^***	9.488	5.661	1.070	3.007a	-
***P* value**	0.023*	0.129*	0.784	0.391	-
**IL-6, %**					
1st	5.9%	24.3%	22.5%	23.5%	25.3
2nd	35.3%	20.0%	23.4%	26.8%	25.8
3rd	35.3%	27.1%	27.9%	21.6%	24.2
4th	23.5%	28.6%	26.1%	28.1%	24.7
*χ^2^*	3.882	1.370	0.994	0.976	-
***P* value**	0.275	0.713	0.803	0.807	-
**IL-12, %**					
1st	23.5%	18.6%	25.5%	27.5%	25.2%
2nd	17.6%	20.0%	31.1%	21.5%	26.6%
3rd	41.2%	30.0%	21.7%	23.5%	24.9%
4th	17.6%	31.4%	21.7%	27.5%	23.3%
*χ^2^*	2.409	4.229	1.076	2.169	-
***P* value**	0.492	0.238*	0.783	0.538	-
**IL-21, %**					
1st	11.8%	22.9%	26.1%	23.5%	25.0%
2nd	23.5%	14.3%	29.7%	27.5%	24.7%
3rd	23.5%	41.4%	19.8%	26.8%	23.1%
4th	41.2%	21.4%	24.3%	22.2%	27.1%
*χ^2^*	2.334	11.221	1.546	2.030	-
***P* value**	0.506	0.011*	0.672	0.566	-

N=728. Inflammatory markers levels are as follows: 1st quartile, 2nd quartile, 3rd quartile, 4th quartile. “*” indicate significant differences between frailty subtypes and inflammatory marker entering multinomial logistic regression models. Variables with missing values: IL-12 (16/728; 2.2%), TGF-β (3/728; 0.4%).

In the multinomial logistic regression models (Table 4), an increase in Tfh2 cells was shown to result in a corresponding decrease in the risk of being cognitive and functional (aOR = 0.92; 95% CI, 0.86–0.99) and physiologically frail (aOR = 0.92; 95% CI, 0.87–0.98). A greater risk of being multi-frail and physiologically frail was associated with a low percentage of Tfh1 (aOR = 0.77; 95% CI, 0.60–0.99; aOR = 0.87; 95% CI, 0.79–0.96) and Tfh17 (aOR = 0.79; 95% CI, 0.65–0.96; aOR = 0.86; 95% CI, 0.78–0.94) cells, respectively. Moreover, we found that participants in the 2nd tertile for TGF-β and IL-12 levels were significantly less likely to be psychologically frail and physiologically frail (adjusted aOR = 0.37; 95% CI, 0.16–0.88; adjusted aOR = 0.40; 95% CI, 0.17–0.97, respectively) compared with those in the top tertile after adjusting for potential confounders.

**Table 4:** Multivariable logistic regression analysis between frailty subtypes (“Relatively healthy” as reference) and biomarker.

**Frail subtypes**	**Biomarker**	**Unadjusted OR 95% CI**	**Adjusted OR 95% CI**
**Multi-frail**	**Tfh cell**	0.89(0.79-1.01)	0.93(0.76-1.14)
	**Tfh1 cell**	0.86(0.73-1.02)	0.77(0.60-0.99) *
	**Tfh2 cell**	0.90(0.82-0.99) *	0.94(0.83-1.06)
	**Tfh17 cell**	0.85(0.73-0.98) *	0.79(0.65-0.96) *
	**TGF-β (pg/mL)**		
	1st	0.00 (0.00-0.00)	0.00 (0.00-0.00)
	2nd	0.57(0.13-2.54)	0.30(0.04-2.19)
	3rd	0.35(0.07-1.82)	0.00 (0.00-0.00)
	4th (Reference)	-	-
	**IL-12 (pg/mL)**		
	1st	1.51(0.12-21.41)	1.47(0.02-109.55)
	2nd	3.01(0.39-23.24)	3.60(0.10-124.92)
	3rd	3.79(0.76-18.92)	9.93(0.73-136.07)
	4th (Reference)	-	-
	**IL-21 (pg/mL)**		
	1st	0.21(0.02-2.95)	0.16(0.00-11.15)
	2nd	0.36(0.06-2.07)	0.17(0.00-6.55)
	3rd	0.33 (0.07-1.62)	0.23(0.02-3.23)
	4th (Reference)	-	-
**Cognitive and functional frail**	Tfh cell	0.96(0.91-1.02)	0.96(0.90-1.02)
	Tfh1 cell	0.97(0.87-1.09)	0.96(0.85-1.09)
	Tfh2 cell	0.94(0.88-0.99) *	0.92(0.86-0.99) *
	Tfh17 cell	0.96(0.87-1.06)	0.95(0.85-1.06)
	**TGF-β (pg/mL)**		
	1st	0.54(0.21-1.36)	0.71(0.24-2.10)
	2nd	0.71(0.31-1.64)	0.63 (0.25-1.59)
	3rd	0.64(0.27-1.47)	0.69(0.25-1.94)
	4th (Reference)	-	-
	**IL-12 (pg/mL)**		
	1st	0.59(0.19-1.84)	0.88(0.24-3.28)
	2nd	0.60(0.22-1.61)	0.59(0.18-1.88)
	3rd	1.24(0.54-2.89)	1.80(0.66-4.88)
	4th (Reference)	-	-
	**IL-21 (pg/mL)**		
	1st	2.13(0.65-6.97)	1.141(0.29-4.56)
	2nd	1.02(0.35-2.97)	0.73(0.22-2.50)
	3rd	2.20(0.92-5.24)	1.77(0.65-4.83)
	4th (Reference)	-	-
**Psychologically frail**	**Tfh cell**	1.02(0.98-1.07)	1.02(0.97-1.07)
	**Tfh1 cell**	0.97(0.87-1.08)	0.97(0.86-1.10)
	**Tfh2 cell**	0.99(0.93-1.05)	1.01(0.94-1.08)
	**Tfh17 cell**	1.02(0.92-1.10)	1.02(0.91-1.13)
	**TGF-β (pg/mL)**		
	1st	0.74(0.34-1.59)	0.62 (0.27-1.42)
	2nd	0.57(0.27-1.23)	0.373 (0.159-0.875) *
	3rd	0.75(0.36-1.56)	0.50 (0.22-1.14)
	4th (Reference)	-	-
	**IL-12 (pg/mL)**		
	1st	0.55(0.20-1.49)	0.48 (0.16-1.44)
	2nd	0.79(0.33-1.86)	0.75(0.29-1.91)
	3rd	0.95(0.43-2.11)	0.98(0.40-2.40)
	4th (Reference)	-	-
	**IL-21 (pg/mL)**		
	1st	1.88(0.66-5.35)	2.76 (0.86-8.92)
	2nd	1.82(0.75-4.42)	2.51(0.91-6.95)
	3rd	1.29(0.56-2.95)	1.70(0.67-4.28)
	4th (Reference)	-	-
**Physiologically frail**	**Tfh cell**	0.99(0.95-1.03)	0.98(0.94-1.03)
	**Tfh1 cell**	0.88(0.81-0.96) *	0.87(0.79-0.96) *
	**Tfh2 cell**	0.93(0.88-0.97) *	0.92(0.87-0.98) *
	**Tfh17 cell**	0.87(0.80-0.94) *	0.86(0.78-0.94) *
	**TGF-β (pg/mL)**		
	1st	1.12(0.55-2.29)	1.46(0.67-3.18)
	2nd	1.56(0.80-3.08)	1.36(0.65-2.83)
	3rd	0.85(0.41-1.74)	0.90(0.41-1.96)
	4th (Reference)	-	-
	**IL-12 (pg/mL)**		
	1st	0.51(0.21-1.23)	0.58 (0.23-1.50)
	2nd	0.38(0.17-0.85) *	0.40(0.17-0.97) *
	3rd	0.61(0.29-1.25)	0.77(0.35-1.69)
	4th (Reference)	-	-
	**IL-21 (pg/mL)**		
	1st	1.59 (0.60-4.23)	1.30(0.46-3.69)
	2nd	2.09(0.91-4.79)	1.84(0.76-4.47)
	3rd	1.96 (0.93-4.14)	1.79(0.80-3.99)
	4th (Reference)	-	-

N=728. Abbreviations: OR: Odds ratio; CI: confidence interval; *P < 0.05.

## DISCUSSION

With improved life expectancy, frailty, a well-established risk factor for poor outcomes, has emerged as a major public health challenge, particularly over the last two decades. Based on frailty is a dynamic process which could be delayed or even reversed, it is necessary to explore targeted biomarkers in frailty. In this study, we comprehensively assessed biomarker associations with objectively determined frailty in the older Chinese population through an adjusted model approach. To the best of our knowledge, our study is the first to confirm the importance of a sex-specific Tfh2 cell biomarker and inflammatory marker TGF-β in older people of different frailty status. We further explored the association between Tfh cell subsets markers, inflammatory markers and frailty subtypes. Our findings provided novel evidence supporting promising biomarker targets in older adults who will benefit from the evaluation of frailty.

In our study, we employed two frailty models, including the mFI model and frailty subtypes, to investigate sex differences in frailty status and different aspects of frailty in the older Chinese population. With regard to frailty status, we reported that frailty affected approximately 15% of subjects >65 years and pre-frailty affected approximately 50% of subjects >65 years, which was in concordance with the findings of previous cross-sectional studies [34], indicating the importance of identifying biomarkers to enable intervention in the earlier stages of physical aging (prefrail status). Our data also showed that females have higher frailty scores than males in community-dwelling populations similarly to previous reports [32], which supported sex-related differences. For frailty subtypes, unlike a previous study that captured physical frailty based on the frailty phenotype [5], we explored firstly the potential subtypes of frailty among older Chinese subjects by applying a validated mFI tool containing multiple components. Latent Class Analysis (LCA) identified four distinct frailty subtypes clustering with different functional components, these comprised multi-frail (all components), cognitive and functional frail (functional activities and cognitive function), psychologically frail (mental state) and physiologically frail (general health status and symptoms), which better reflected the multisystem and multidimensional nature of the biological changes involved in frailty. Moreover, we examined the inter-relation of the frailty status and frailty subtypes in our study. The results showed that the proportion of relatively unhealthy individuals (48.4%) were lower than proportion of pre-frail and frail individuals (68%). As mFI increased, following states were predominant in successive order: relatively heathy, physiologically frail, psychologically frail, cognitive and functional frail, and multi-frail.

Investigation of T cell subsets, and its dysregulation of the immunesenescence in frailty lags behind similar research into ageing processes [11, 35]. Little information is available about potential Tfh alterations in frailty and the importance of Tfh cell subsets in frailty was first demonstrated based on findings from two frailty models. These findings suggested that significant sex-specific Tfh cell subset dysregulation is considered to be responsible, at least in part, for frailty. However, inverse or inconsistent relationships were observed with Tfh cell subset compartments, particularly for Tfh2 cells in both frailty measures. We found the opposite trend to that expected for Tfh2 cells with regard to frailty status in females, which was inconsistent with the general acceptance that CD4^+^ T cells decline with age and frailty [11, 36]. However, subsequent analysis showed that a higher Tfh2/Tfh17 cell ratio was more likely to be associated with a lower frailty level, suggesting that subset skew may be involved in the incidence of frailty. Combining these two cellular markers might constitute a more robust biomarker for pre-frail or frail diagnosis than using a single marker. Additionally, an association between the Tfh2/Tfh17 cell ratio and frailty was only found in females, which suggests that detecting frailty based on markers may be more effective for women than for men [37]. Little is known regarding the role of the three Tfh cell subsets and this might be due to the fact that Tfh cells mainly play an important role in protective immunity against pathogens and autoimmune disease [14]. Therefore, in this study, Tfh cell subsets were analyzed in terms of three frailty states comprising seven dimensions, which included the general health status, functional activities and mental state, to determine their role in the pathogenesis of frailty. This speculation was confirmed by the negative association between all Tfh cell subset compartments and the physiologically frail subtypes. Taking into account the limited sample size, no significant difference was found between the different sexes and the frailty subtypes, which implied that the subtypes might be distributed across a homogeneous population. Interestingly, we also found that specific Tfh cell subsets were associated with different subtypes, mainly for the cognitive and functional frail and the multi-frail groups. Based on theory of immunosenescence, we consider that effects of specific deregulations in the T cell pool is indicators of dysfunctional immune system in frailty, which suggests special combination of Tfh cell subsets skew mediates the development of different types frailty. These findings helped explain the role of immunosenescence in frailty and suggested that the Tfh subset may be one of the main pathophysiological mechanisms underlying frailty.

It is conceivable that B cells might lead to aging-associated immune decline and little evidence was found in frailty [38]. As expected, we demonstrated that a significant decrease in B cells correlated with the severity of the frailty status in both females and males, which was consistent with the observations of Nevalainen et al [39] who reported a correlation between sex and CD19^+^ B cells in older individuals (90+ years) without adjusting confounders. However, there was no significant association between frailty subtype and the different sexes in multivariable analyses in the present study. It may be that the balance of B cell subsets, not just the B cell percentage, affects frailty subtypes, and this requires further analysis in future studies.

Inflammaging, one distinct component of immunosenescence, has increasingly being recognized to be an underlying mechanism of frailty [40]. We examined not just the conventional inflammatory biomarkers (IL-6) in frailty development, but also explore other inflammatory biomarkers related to immunosenescence which might play an important role in frailty development. There are some inconsistencies in the previously reported data regarding the association between inflammatory markers and frailty [28]. In our study, we firstly reported that a high level of TGF-β is associated with the severity of frailty in females only, demonstrating a strong female-specific correlation in older individuals. Similarly, no significant association was detected between any of the inflammatory markers and the risk of frailty in men in a longitudinal study of ageing in England [41]. It has also been reported that participants in the 2^nd^ tertile of TGF-β were more associated with psychological frailty compared with those in the top tertile and the importance of TGF-β in frailty has been reinforced by consistent findings across two frailty models. Our results updated the evidence of involvement of TGF-β alterations in frailty status and subtype in older adults, beyond that related to aging previously [42]. In addition, the inflammatory marker IL-12 was demonstrated to have a positive association with physiological frailty in one study, but this was not consistent across studies [24]. This inconsistency might be because the frailty evaluation was based on physical frailty and sarcopenia, which is defined by physical impairment and muscle loss [43]. There is strong evidence to suggest that the IL-6 level is a key pathophysiological factor in frailty. However, our data are not consistent with findings from previous cross-sectional frailty studies. It is possible that these differences are due to the different frailty measures or techniques employed to measure biomarkers and age differences. Additionally, in previous reports, no relationship was found between higher IL-6 levels and frailty in a longitudinal study using the frailty phenotype approach [30, 44] and more studies are needed to explore the role of Il-6 in frailty using a large sample. In summary, controlling high levels of inflammatory markers (TGF-β and IL-12 in particular) earlier might be of benefit when trying to limit the development of frailty.

Several limitations of our study must be acknowledged. First, based on our limited sample size, we were unable to fully explore sex-related differences with regard to frailty subtypes. Our study showed the effectiveness of applying the FI scale developed in the older Chinese population as a measure of frailty, but caution needs to be taken regarding the generalizability of our results to the populations of other countries. In addition, we were unable to draw conclusions regarding the causality and predictive effects between the Tfh cell subsets, inflammatory biomarkers and frailty because of the cross-sectional design of our study. Longitudinal studies are therefore warranted in the future. Finally, although we established strict exclusion criteria, we cannot completely eliminate the possibility that other infections could have affected the expression of biomarkers.

In conclusion, we report cross-sectional associations between Tfh cell subsets and inflammatory biomarkers simultaneously with the frailty status in a sex-specific manner in the community-dwelling, older, Chinese population. Our study provides an overview of inflammation and immune senescence in frailty subtypes and ascertains the additional value of these subgroups for developing efficient intervention strategies. Tfh cell subsets and inflammatory biomarkers were more closely associated with frailty in women than in men. Thereby, targeted intervention to prevent frailty should be frequently recommended for women. Our work also highlights the role of Tfh cells in the physical decline of older subjects and suggests that interventions targeted toward increasing Tfh cell subsets levels might be beneficial for older adults with physiologically frailty. Finally, the potential predictive effects of Tfh cell subsets, and TGF-β and IL-12 as biological markers of frailty, in the vulnerable older population should be further investigated in prospective studies.

## MATERIALS AND METHODS

### Study population

From October 2017 to June 2019, a cross-sectional study was conducted in population-based community-dwelling individuals using a random cluster sampling method from 21 selected communities (villages) in Dalang Town, Dongguan, China. We interviewed 5341 subjects to conduct frailty assessment and further selected 4 communities (villages) including 892 individuals aged >60 years using a random cluster sampling method from 21 selected communities (villages) to biological testing during on-site physical examinations. And 864 subjects (97%) consented to biological testing. Then 728 individuals aged >60 years were finally included due to the exclusion of subjects for whom immune phenotype (n=136) were missing because of blood sample problem and inappropriate experiment process in our study. A questionnaire, in the form of a face-to-face interview, was conducted to collect baseline characteristics, including social demographic data (sex, age, education) and unhealthy behaviors (smoking). The inclusion criteria were individuals aged >60 years who agreed to be involved in the study. The exclusion criteria were an acute inflammatory autoimmune disease, Alzheimer's disease, a disability causing the subject to be bedridden and an inability to communicate adequately. This study was approved by the Ethics Committee of the Affiliated Hospital of Guangdong Medical University (Permit Number: YJYS2018046) and written informed consent was provided by individual participants.

### Frailty measurement variables

The frailty measurement variables in this study were based on the frailty evaluation scale previously developed by the project team in the early stages following standard procedures, and contained seven dimensions and a total of 33 items. The general health status contained six items, including “Are you in poor health now?” and “Has your health deteriorated compared with 1 year ago?” Activities of daily living contained nine items, which included “In the past month, did you need help to complete the following activities: bathing, dressing, etc.?” Functional activities contained six items, including “In the past month, did you need help to complete the following activities: going up or down stairs, shopping, etc.?” Symptoms contained four items, including “Have you had any physical pain in the past month?” and “Is your vision impaired?” Mental state contained six items, including “Have you had the following feelings in the past week: finding it hard to concentrate, feeling sad or depressed, etc.?” Social support contained one item: “Are you living alone?” For the 32 questions indicated above, the respondent only needed to answer “Yes” or “No” according to their own circumstances. In addition, cognitive function was assessed using the Mini-Mental State Examination (MMSE) and the investigator evaluated whether the respondents' cognitive function was poor based on their final score. A score of 17 points or fewer was marked as “Yes”, and a score of more than 17 points was marked as “No”.

### Processing of blood samples

Fasting venous blood samples (~4 mL) were collected from eligible subjects. The serum was obtained (500 μL/tube) and stored at −80°C. The remaining blood samples were used for preparing peripheral blood mononuclear cells (PBMCs) by density gradient centrifugation. Whole blood was diluted with an equal volume of RPMI 1640 medium (Gibco, Grand Island, USA) and then added to a SEPMATE-15 tube (StemCell Technologies, Vancouver, BC, Canada) containing density gradient medium (Lymphoprep) (StemCell Technologies) and was centrifuged at 1200 × *g* for 10 min. The supernatant enriched for PBMCs was collected and washed twice with an equal volume of RPMI 1640 medium at 300 × *g* for 10 min.

### Flow cytometry

Human freshly-isolated PBMCs at 10^6^/tube for each individual were stained with antibodies specific for T and B cells. Cells were then surface stained with a mixture of the following mouse anti-human antibodies (BD Biosciences): Hu CD3 PE HIT3a, Hu CD19 PE-Cy7 SJ25C1, Hu CD196 (CCR6) BV421 11A9, Hu CD183 APC 1C6/CXCR3, Hu CXCR5 (CD185) BV510 RF8B2 and Hu CD4 FITC RPA-T4. These samples were incubated at room temperature for 30 min, protected from the light. Cells were washed twice and fixed with 2% paraformaldehyde until acquisition on a BD LSRFortessa.

### Determination of TGF-β, IL-6, IL-12 and IL-21 levels by ELISA

The levels of TGF-β, IL-6, IL-12 and IL-21 in plasma samples were quantitatively determined using the relevant human ELISA kits (ZCI BIO, Shanghai, China) according to the instructions provided by the manufacturer and all standards were tested in duplicate.

### Statistical analysis

### Frailty classification

### Frailty status according to the modified frailty index

In our previous study, a frailty evaluation scale was developed based on a FI model [1]. The mFI was calculated from the 33 health deficits detailed above. Given that assessment of frailty status is based on total score of 33-item, a stricter standard that we chose deletion method instead of imputing missing data values for frailty status would be established when missing value existed. The number of health problems (answering “yes”) for each subject was divided by the total number of health problems (n=33) and the scores ranged from 0 to 1, with a higher score indicating a higher level of frailty. The scale has previously been tested in the older Chinese population and its reliability and validity have been proven [3]. Participants with an mFI<0.08, 0.08≤mFI<0.22 and mFI≥0.22 were classified into three frailty states: non-frail, pre-frail, and frail, respectively.

### Frailty subtypes by latent class analysis

We used LCA to explore the latent classes of frailty using the 33 variables mentioned above for the frailty assessment [45]. When few missing values exist (5%), samples including missing values would be retained, which did not affect definition of frailty subtype [46]. Briefly, the best class solution was identified to explain the association among a set of observed variables with the least number of latent classes, and further to cluster similar individuals if the *P* values of the two values achieved a significant level (*P*<0.01). The LMR and the BLRT were used to compare the differences in fit of the latent class models. According to the differences in the conditional probability distribution and the characteristics of the observed variables, we interpreted and named each latent class. The details of the frailty subtype measurements are shown in the Supplementary Materials and Methods.

### Data preparation and descriptive analysis

Demographic characteristics are described using the means ± standard deviations (SD) or the medians with interquartile ranges (IQRs) for continuous variables (mFI), whereas the frequencies (%) of the population (skewed variables) were used for categorical variables (age, sex, education, smoking, inflammatory markers, frailty status, frailty subtypes). Differences in baseline characteristics between frailty groups were assessed by Pearson’s *χ^2^* test (categorical variables).

### Analysis of immune biomarkers associated with frailty status and frailty subtypes

We performed a univariate analysis using the *χ^2^* test (categorical variables) and the Kruskal–Wallis test (continuous variables). Then, immune biomarker variables with *P*<0.25 and interaction terms with *P* <0.05 in the univariate analyses were evaluated further for inclusion in the final ordinal or multivariable disordered multi-class logistic regression to obtain OR (frailty status or frailty subtypes as dependent variable) and aOR (age, sex, education, smoking) and their 95% CI. When necessary, quartiles of inflammatory markers were used for the *χ^2^* test and logistic regressions [47]. The highest quartile was used as the reference category. For the logistic regression, *P*≤0.05 was considered to indicate a statistically significant difference. The strength of the multicollinearity was examined using the variance inflation factor (VIF) and VIF>10 indicated that the model exhibited multicollinearity. The analysis was carried out using SPSS software version 20.0.0 (SPSS Inc., Chicago, IL, USA). Figures were prepared using STATA version 12.0 (Stata Corp., College Station, TX, USA) and GraphPad Prism version 8.0 (GraphPad Software, San Diego, CA, USA).

## Supplementary Material

Supplementary Materials

Supplementary Tables
