# Supplementary material for: Tfh cell subset biomarkers and inflammatory markers are associated with frailty status and frailty subtypes in the community-dwelling older population: a cross-sectional study
Source: Aging (Albany NY). 2020 Feb 8;12(3):2952–73. doi: 10.18632/aging.102789 (PMC7041730; doi:10.18632/aging.102789)
Supplement: Supplementary Materials [file aging-12-102789-s001..pdf]

SUPPLEMENTARY MATERIALS

Supplementary Materials and Methods

Frailty subtypes by latent class analysis (LCA)

Latent class analysis (LCA)

First, we used latent class analysis (LCA) to explore the latent classes of frailty. LCA is a statistical technique for exploring the categorical latent variables behind the statistically related categorical observed variables; it combines latent variable theory with categorical variables. Based on the observed variables, the purpose of LCA is to find the best class solution, that is, to explain the association among a set of observed variables with the least number of latent classes, and further to cluster similar individuals. The observed variables we used in the LCA were the 33 variables mentioned above for the frailty assessment.

In this study, the maximum likelihood (ML) method was used for parameter estimation, and the expectation-maximum (EM) method was used in the iterative process. We fitted 1–7 potential category models respectively. In the evaluation of the models, the indicators included the Akaike information criterion (AIC), Bayesian information criterion (BIC), and adjusted Bayesian information criterion (aBIC). In general, the smaller the numerical values of these indicators, the better the model fits. The entropy index was used to evaluate the accuracy of the classification. The Lo–Mendell–Rubin (LMR) and the bootstrap-based likelihood ratio test (BLRT) were used to compare the differences in fit of the latent class models; if the *P* values of the two values achieved a significant level ($P < 0.01$), this indicated that the model with K classes was significantly better than the model with K-1 classes. We determined the final classification based on comprehensive consideration of the above indicators. The LCA analysis was done using Mplus 7.4.

Naming of latent classes

The probability of each latent class and the conditional probability of each observed variable under the latent classes were estimated by the EM method based on the selected classes. Then, according to the differences in the conditional probability distribution and characteristics of the observed variables, we interpreted and named each latent class.

SupplementaryResults

Naming of latent classes

According to the conditional probability distribution differences and characteristics of 33 items (Table 3), each latent class could be named. Compared with the other four classes, class 5 had the highest probability of answering “no” to all items, indicating that the group’s overall health was good, so class 1 was named “relatively healthy.” Conversely, class 1 had the highest probability of answering “yes” to most of the items, indicating that the group’s overall health status was poor, so class 1 was named “multi- frail”. The conditional probabilities of class 2, class 3 and class 4 answering “yes” to most of the items were between those of class 1 and class 5. Further observations showed that class 2 had a higher probability of performing poorly on items representing functional activities (items 16–21) and cognitive function (item 33), so class 2 was named “cognitive and functionally frail”. Similarly, class 3 had a higher probability of performing poorly on items representing mental state (items 26–31), so class 3 was named “psychologically frail”, and this group had the highest probability of physical pain and poor sleep. Class 4 was named “physiologically frail”, because the health problems of this group were mainly concentrated on the components representing general health status and symptoms.
