# Supplementary material for: Tfh cell subset biomarkers and inflammatory markers are associated with frailty status and frailty subtypes in the community-dwelling older population: a cross-sectional study
Source: Aging (Albany NY). 2020 Feb 8;12(3):2952–73. doi: 10.18632/aging.102789 (PMC7041730; doi:10.18632/aging.102789)
Supplement: Supplementary Tables [file aging-12-102789-s002..pdf]

SUPPLEMENTARY TABLES

Supplementary Table 1. Fitting statistical results of the latent class model.

Model	k	AIC	BIC	aBIC	Entropy	LMR	BLRT
1C	33	18217.318	18368.798	18264.012	_	_	_
2C	67	16414.965	16722.515	16509.769	0.971	0.0075	<0.001
3C	101	15943.216	16406.837	16086.131	0.807	0.2241	<0.001
4C	135	15578.729	16198.420	15769.753	0.85	0.1142	<0.001
5C	***169***	***15375.308***	***16151.069***	***15614.441***	***0.865***	***<0.001***	***<0.001***
6C	203	15291.157	16222.989	15578.401	0.872	0.3886	<0.001
7C	237	15257.239	16345.141	15592.592	0.836	0.1227	<0.001

Abbreviations: AIC, Akaike information criterion; BIC, Bayesian information criterion; aBIC, adjusted Bayesian information criterion; LMR, The Lo–Mendell–Rubin; BLRT, the bootstrap-based likelihood ratio test.

Supplementary Table 2. Conditional probability distribution of observed variables under the five latent classes.

Items	Class1	Class2	Class3	Class4	Class5
**General health status**					
1.Are you in poor health now?					
No	0	0.234	0.098	0.27	0.474
Yes	1	0.766	0.902	0.73	0.526
2. Has your health deteriorated compared to Yes year ago?					
No	0.471	0.591	0.717	0.633	0.894
Yes	0.529	0.409	0.283	0.367	0.106
3. Have you been to the hospital in the past year?					
No	0.294	0.499	0.602	0.506	0.723
Yes	0.706	0.501	0.398	0.494	0.277
4. Did you fall in the past year?					
No	0.823	0.795	0.869	0.875	0.92
Yes	0.177	0.205	0.131	0.125	0.08
5. Is the BMI at normal level?					
No	0.53	0.661	0.623	0.453	0.595
Yes	0.47	0.339	0.377	0.547	0.405
6. Do you have any chronic diseases now?					
No	0.236	0.223	0.419	0.192	0.417
Yes	0.764	0.777	0.581	0.808	0.583
**Activities of daily living** (In the past month, did you need help to complete the following activities?)					
7. Bathing					
No	0.176	1	1	1	1
Yes	0.824	0	0	0	0
8. Dressing					
No	0.234	1	1	1	1
Yes	0.766	0	0	0	0
9. Eating					
No	0.176	1	1	1	1
Yes	0.824	0	0	0	0
10. Cooking					
No	0	0.916	1	1	0.992
Yes	1	0.084	0	0	0.008
11. Washing					
No	0.058	0.958	1	0.994	1
Yes	0.942	0.042	0	0.006	0
12. Walking around the house					
No	0.176	0.944	1	0.994	1
Yes	0.824	0.056	0	0.006	0
13. Defecation control					
No	0.118	0.972	1	1	1
Yes	0.882	0.028	0	0	0
14. Toileting					
No	0.118	0.986	1	1	1
Yes	0.882	0.014	0	0	0
15. Dressing up					
No	0.234	0.931	1	1	1
Yes	0.766	0.069	0	0	0
**Functional activity** (In the past month, did you need help to complete the following activities?)					
16. Up or down stairs					
No	0.236	0.527	1	0.977	0.988
Yes	0.764	0.473	0	0.023	0.012
17. Shopping					
No	0.059	0.451	1	0.985	1
Yes	0.941	0.549	0	0.015	0

18. Calling					
No	0.176	0.407	0.956	0.931	0.958
Yes	0.824	0.593	0.044	0.069	0.042
19. Walking far distance					
No	0.059	0.207	1	1	0.976
Yes	0.941	0.793	0	0	0.024
20. Going out alone					
No	0.059	0.085	0.892	0.958	0.946
Yes	0.941	0.915	0.108	0.042	0.054
21. Stooping to pick up something on the ground					
No	0.177	0.267	0.725	0.946	0.919
Yes	0.823	0.733	0.275	0.054	0.081
**Symptom**					
22. Have you had any physical pain in the past month?					
No	0.236	0.253	0.2	0.323	0.602
Yes	0.764	0.747	0.8	0.677	0.398
23. Is your vision impaired?					
No	0.412	0.655	0.598	0.16	0.922
Yes	0.588	0.345	0.402	0.84	0.078
24. Is your hearing impairment?					
No	0.471	0.646	0.764	0.305	0.956
Yes	0.529	0.354	0.236	0.695	0.044
25. Have you had a poor sleep in the past month?					
No	0.294	0.447	0.251	0.511	0.679
Yes	0.706	0.553	0.749	0.489	0.321
**Mental state** (Have you had the following feelings in the past week?)					
26. Hard to concentrate					
No	0.589	0.731	0.61	1	0.989
Yes	0.411	0.269	0.39	0	0.011
27. Feeling Sad or depressed					
No	0.353	0.655	0.209	0.885	0.932
Yes	0.647	0.345	0.791	0.115	0.068
28. Feeling lonely					
No	0.412	0.736	0.482	0.998	0.99
Yes	0.588	0.264	0.518	0.002	0.01
29. Memory was worse than peers					
No	0.471	0.672	0.428	0.637	0.892
Yes	0.529	0.328	0.572	0.363	0.108
30. Didn't like going out (connecting with people)					
No	0.707	0.845	0.775	0.965	0.973
Yes	0.293	0.155	0.225	0.035	0.027
31. Feeling tired and not in spirit					
No	0.648	0.835	0.825	0.957	0.981
Yes	0.352	0.165	0.175	0.043	0.019
**Social support**					
32. Are you living alone.					
No	0.647	0.701	0.842	0.834	0.934
Yes	0.353	0.299	0.158	0.166	0.066
**Cognitive function**					
33. Poor cognitive function					
No	0.352	0.56	0.935	0.824	0.919
Yes	0.648	0.44	0.065	0.176	0.081
Latent classes probability	0.023	0.098	0.152	0.210	0.516
Sample frequency	17	71	111	153	376
